# Rebuilding Body–Brain Interaction from the Vagal Network in Spinal Cord Injuries

**DOI:** 10.3390/brainsci11081084

**Published:** 2021-08-18

**Authors:** Maria Luisa De Martino, Mina De Bartolo, Erik Leemhuis, Mariella Pazzaglia

**Affiliations:** 1 Department of Psychology, Sapienza University of Rome, Via dei Marsi 78, 00185 Rome, Italy; demartinomarialuisa@gmail.com (M.L.D.M.); debartolo.mina@gmail.com (M.D.B.); erik.leemhuis@uniroma1.it (E.L.); 2 Body and Action Lab, IRCCS Fondazione Santa Lucia, Via Ardeatina 306, 00179 Rome, Italy

**Keywords:** vagus nerve, taVNS, spinal cord injury, body awareness, interoceptive signals, pain, neuroplasticity

## Abstract

Spinal cord injuries (SCIs) exert devastating effects on body awareness, leading to the disruption of the transmission of sensory and motor inputs. Researchers have attempted to improve perceived body awareness post-SCI by intervening at the multisensory level, with the integration of somatic sensory and motor signals. However, the contributions of interoceptive-visceral inputs, particularly the potential interaction of motor and interoceptive signals, remain largely unaddressed. The present perspective aims to shed light on the use of interoceptive signals as a significant resource for patients with SCI to experience a complete sense of body awareness. First, we describe interoceptive signals as a significant obstacle preventing such patients from experiencing body awareness. Second, we discuss the multi-level mechanisms associated with the homeostatic stability of the body, which creates a unified, coherent experience of one’s self and one’s body, including real-time updates. Body awareness can be enhanced by targeting the vagus nerve function by, for example, applying transcutaneous vagus nerve stimulation. This perspective offers a potentially useful insight for researchers and healthcare professionals, allowing them to be better equipped in SCI therapy. This will lead to improved sensory motor and interoceptive signals, a decreased likelihood of developing deafferentation pain, and the successful implementation of modern robotic technologies.

## 1. Introduction

The maintenance of homeostatic stability in the body necessitates balancing the internal visceral feedback in relation to the environmental context. This, in turn, facilitates the identification of two levels of control as follows: proper interoceptive inference and allostatic interoceptive inference. In the former, during extremely low oxygen or high temperature, for example, the visceral motor and sensory signals permit the maintenance of homeostasis through simple reflex arcs [1,2]. The brain can directly involve adaptive physiological reflexes, thus increasing the respiratory rate or decreasing the systolic blood pressure [2]. The integrity of the system is maintained through predictive interoceptive mechanisms that regularly compare with visceral inputs, thereby allowing the former to be constantly updated, in relation to the state of the body [3,4,5,6]. Allostatic interoceptive inferences intervene for reflex mechanisms that are insufficient to maintain the physiological parameters in the adaptive range. Thus, the first considered low-level processes adjust to the demands of the external environment [2,7,8].

Patients with SCI are an example of a clinical population with a disturbance in their homeostatic balance. In addition to an interruption of sensory–motor connections, they experience a loss of somatic and visceral information that reaches the brain, depending on the level of spinal cord lesion. This results in a misperception of the interoceptive signals, thus contributing to the typical distortions of body awareness, and the internal conscious representation of the body [9,10].

Interestingly, the vagal pathway is not involved in spinal lesions. Briefly, the vagus nerve represents a homeostatic key component of the parasympathetic branch of the autonomic nervous system (ANS) and carries ascending interoceptive sensory information about internal organs and the enteric nervous system. Through vague, visceral and interoceptive signals—particularly respiratory and cardiac signals—this information reaches the superior cerebral areas by one of the major brainstem interoceptive targets, the nucleus tractus solitarius (NTS). It is fundamental for controlling physiological states and is principally connected via the thalamus to several interoceptive areas, such as the somatosensory cortex and insula [11,12,13]. These areas play an important role in bodily representation and the sense of body ownership. Therefore, non-invasive vagal stimulation (taVNS)—a technique currently applied in the treatment of various disorders [14,15]—can be supposedly used to modulate interoceptive signals and, consequently, the sense of body awareness. We consider a rehabilitation perspective that not only focuses on building a sense of awareness of the external body (through visual, proprioceptive, and somatomotor feedback [10]), but also attempts to reconstruct the internal sense of body consciousness. The inclusion of taVNS in rehabilitation training that uses robotic assistive tools for movement (i.e., exoskeletons) can be beneficial. We present some evidence to support the role of this stimulation in promoting neuroplasticity and modulating interoceptive signals, thus highlighting the centrality of the latter (particularly the respiratory signals) in establishing a better sense of embodiment.

## 2. Rehabilitation Challenge: Can an Interoceptive Process Shape Body Awareness?

### 2.1. Losing the Body Following SCI

Patients with SCI present with an interruption of the sensorial, proprioceptive, and motor information originating from the limbs as well as some somatic–visceral pathways. The degree of impairment depends on the level and completeness or otherwise of the lesion. Sensorimotor deficits represent a relevant source of disability in these patients. However, the difficulties of the interoceptive domain deserve attention [16,17]. Significant respiratory, gastrointestinal, and cardiovascular injuries are a common consequence of high-level SCI (sympathetic innervation exits at the T1–5 level). In addition, it affects the urinary system, skeletal muscles, bones, soft tissues, and spleen. Gastrointestinal dysfunctions, including severe constipation, incontinence, abdominal pain, and a deaf sensation in the abdomen, highlight the difficulty in perceiving internal signals in these patients [9,18]. Moreover, the hypothalamic connection to the spinal sympathetic circuits is substantially impaired, and individuals with lesions above T6 can manifest hypothermia. Individuals with lesions above T6 also present with significant respiratory system deficits that manifest as respiratory failure, therefore causing severe difficulty in breathing and coughing. This can be attributed to insufficient lung compliance [16,19]. Furthermore, these individuals experience difficulties in feeling a sense of hunger or thirst, as well as reduced cardiac awareness [20,21].

The condition described above could lead to an altered interoceptive perception, which is profoundly associated with the alterations in body awareness that characterize SCI [10]. These patients experience an altered sense of ownership of their own body, which is expressed in several ways, such as the patients touching parts of their body to assure themselves of their existence. This bodily misperception is explained by the reduction in peripheral inputs (sensory, motor, and interoceptive) reaching the fronto-temporo-parietal and insular multisensory areas, which are responsible for body perception. Patients with SCI—principally those with more severe injuries—experience a strong effect of the rubber hand illusion (RHI) that connects deafferented body parts with a general body representation [20,22].

### 2.2. Looking at Embodiment through Interoceptive Processes

Patients with SCI might be more prone to embody robotic tools, considering their altered sense of body ownership and greater flexibility in body representation [10,23,24].

Particular attention is directed to an embodiment rehabilitation process that allows the inclusion of an assistive tool in body representation through multisensory integration processes to prevent maladaptive neuroplasticity [25,26]. Multisensory integration is a strength of rehabilitation processes and could involve not only sensory (sight, proprioception, and touch) and motor aspects, but also internal body signals. Internal body signals are damaged in SCI; however, they contribute substantially to the sense of bodily consciousness.

The first step in this direction was accomplished by Plotkin et al. [27], who demonstrated that sniffing can achieve the movement control of an electric wheelchair via an “in/out” code, particularly in tetraplegics and “locked-in” patients. If the sniffing process is mediated by the voluntary motor control of the soft palate, which determines a different air pressure in the nasal cavities (mostly exteroceptive aspects), it could be more effective for SCI patients with internal control of inspiration and exhalation. This is because respiratory interoceptive signals are considered to be a bridge between the body and the environment, a means by which the body acts on the outside world. Furthermore, in a patient with SCI who is unable to move intentionally, respiratory processes could represent a means to experience being in control of one’s own actions.

## 3. Interoception: A Way to a Stable and Integrated Sense of Self

Signals from the body are constantly monitored by the brain, which influences a variety of mental processes and complex human behaviors, ranging from feelings in the gut to the heartbeat. Despite the anatomy of the homeostatic neural pathway being relatively well known, the process by which signals from the interoceptive system can shape the body in the brain is not well understood.

Tsakiris et al. [28] discovered a correlation between interoception, exteroception, and the sense of self-consciousness. They measured interoceptive accuracy (IAcc) through a heartbeat detection task in a group of participants who subsequently underwent the RHI procedure. A negative correlation was observed between the IAcc measurement and the RHI result, such that a higher level of IAcc corresponded to a lesser success in RHI. Despite other authors, such as Horváth et al. [29], not replicating these results, further evidence supporting this hypothesis originates from studies on patients with chronic pain and body image alterations.

### 3.1. Interoception and Body Image Disorders

Some classes of patients with body image distortions, such as those with anorexia or other eating disorders, appear to be characterized by low levels of IAcc and interoceptive awareness [30,31,32,33]. Studies on anorexic patients have demonstrated the altered activity of the insula compared to healthy controls [34,35]. Furthermore, Strigo et al. [36] reported on a discrepancy between brain activation during the moment that anticipates pain and the moment of perception of the pain stimulus. The former was characterized by the greater activation of the right anterior insula, dorsolateral prefrontal cortex, and cingulate, while the response to pain was signaled by greater activation in the posterior insula. This depends on an altered ability to perceive the body’s internal signals. Moreover, in other disorders that involve alterations in body image, researchers observed a lower sensitivity to the perception of interoceptive signals, such as in patients with chronic pain [37].

The dysregulation of physiological parameters has been detected particularly in patients with phantom limb pain. It has been implicated in the pathogenesis of chronic pain, including an increase in resting heart rate (HR) and a reduction in heart rate variability (HRV) [38]. Therefore, distortions of the body image can be considered as a possible imbalance between interoceptive and exteroceptive information [30].

### 3.2. Interoceptive Accuracy as a Condition for Exteroceptive Stability

Similar to patients with a low IAcc [28], the RHI produced a stronger effect in patients with eating disorders [39,40], which presented as an altered perception of interoceptive signals. These results can be explained by considering the tendency of the brain to rely on the most stable and precise sensory information. From the perspective of predictive coding, subjects with more stable representations of the internal body (higher level of IAcc) lean on interoceptive information when observing a limb that could be attributed to themselves during the RHI paradigm, despite not feeling it physiologically as one’s own. In contrast, a lower level of IAcc improves the success of the illusion because of the predominant visual–tactile information, thus minimizing the interoceptive prediction error [30,41,42]. In this context, the role of the insula in integrating environmental information and body signals deserves mention, thereby allowing the construction of a unitary sense of self-awareness [6,43,44]. Thus, several researchers have supported this perspective of multisensory integration that involves interoceptive processes [45,46]. For example, by using a virtual cardiac rubber hand, Suzuki et al. [47] combined the visual feedback of the virtual hand with the participants’ heartbeat. The online integration of visual, tactile, and cardiac signals lead to a sense of ownership of the virtual hand. Several studies have also highlighted the importance of the respiratory-related modulation of the sense of body awareness [48]. In synchronous stimulation, the subjects experienced a sensation of dislocation of the breath towards the virtual body. The participants referred the sense of breathing agency to the virtual body, thus feeling a sensation of identification with it [49,50].

In summary, the interoceptive sense of self is critical for maintaining body stability, even in relation to environmental changes, thus modulating the impact of exteroception-driven representations of self [51]. The vagus nerve represents a widespread pathway of homeostatic innervation, directly intervening in the integration of neurovisceral signals from the body [52]. Thus, it seems relevant to explore its role in the process of building an integrated sense of the bodily self.

## 4. The Potential Effects of taVNS on Interoceptive Processes

Recently, several researchers have explored a new potential therapy named vagus nerve stimulation (VNS). VNS was developed as an invasive practice and was approved by the Food and Drug Administration for the treatment of both depression and epilepsy [15].

Furthermore, taVNS has been used for clinical and research purposes and has several advantages over the invasive technique [53,54,55].

### 4.1. Vagal Stimulation and Neuroplasticity

Previous studies have demonstrated that vagal stimulation (both invasive and non-invasive) can be useful in promoting neuroplasticity. It activates various neuromodulatory networks, such as cholinergic, noradrenergic, and serotonergic networks, and promotes the release of fibroblast growth factor [56,57,58]. The involvement of these neuromodulatory systems, together with synaptic eligibility traces, drives synaptic plasticity [59]. Combining rehabilitation (motor-sensory) training with vagal stimulation exerts an effect on the organization of the connectivity of the cortico-spinal tract [60]. This finding has been reported in studies using invasive vagal stimulation in animal and human models following a stroke. For example, studies using rats have demonstrated that tactile therapy along with invasive vagal stimulation can lead to the reorganization of the primary sensory cortex, thus improving sensory function [61]. Similarly, this stimulation allows the better recovery of motor functions—particularly for movements of the upper limbs—if associated with motor training, compared to rehabilitation alone [62,63,64]. The transient brain response evoked by each heartbeat plays a role in cognitive functions that are usually studied separately, such as body perception, self-related cognition, and spatio-temporal evolution of dynamic visual events [65]. In addition, long-term active VNS paired with rehabilitation training can benefit the functional recovery of patients following ischemic stroke [66]. VNS in animal models of spinal or peripheral injury has been demonstrated to improve sensory and motor function of the forelimbs, on combining the stimulation with active rehabilitation training [67,68,69]. A stronger connection has been recorded between the brain and the deafferented muscles, with the possibility of improving the synaptic strength of the spared connections or to create new ones (in continuity with reports on the reinforcement of the connectivity of the corticospinal tract) [67,68]. However, few studies have highlighted the potential of taVNS in promoting synaptic plasticity. These studies are principally conducted in stroke survivors, in whom the association of taVNS with motor rehabilitation or, specifically, with robotic-assisted rehabilitation, can improve forelimb function [70,71,72]. Considering taVNS can improve the mechanisms of the neuroplasticity induction [73,74], a similar effect can likely be observed in patients who present with a brain–body disconnection. However, further studies are needed in this direction.

### 4.2. TaVNS and Pain

TaVNS exerts an effect on pain perception, and its application reportedly increases the threshold of mechanical and pressure pain in healthy subjects [75]. We identified studies on migraines from the most consistent evidence supporting the use of non-invasive vagal stimulation for the treatment of pain. Both non-invasive cervical stimulation and taVNS were applied to such patients, displaying consistent effects in reducing the frequency and severity of migraine attacks [15,76,77,78]. This technique has been applied to the treatment of several other disorders. For example, the application of taVNS synchronized with respiratory rhythm (RAVANS) can reduce chronic pelvic pain due to endometriosis [79].Furthermore, taVNS can improve the sense of fatigue and chronic musculoskeletal pain in patients with systemic lupus erythematosus [80].

Despite no clear definition, the precise mechanism that mediates the analgesic effect of vagal stimulation, especially the non-invasive technique, has been attributed to projections of the auricular branch of the vagus nerve towards the NTS. Through the NTS, the sensory afferences (principally those of type A) exert an indirect effect on the inhibition of the spinal regions involved in the pain process, and on the activity of brain areas typically involved in pain modulation (such as the rostral ventromedial medulla, periaqueductal gray, and anterior cingulate cortex) [54,73,79,81]. Therefore, we hypothesize that taVNS exerts an effect on central pain processing as well as on peripheral nociceptive systems, thus supporting the use of this technique for the treatment of patients with chronic pain [75].

### 4.3. taVNS: A Way to Modulate Interoceptive Processes

Some studies have demonstrated parasympathetic modulation mediated by taVNS, thereby highlighting the effects on the HR rate, blood pressure, and HRV [82,83,84], as well as the effects of vagal stimulation on respiration, particularly for strengthening respiratory sinus arrhythmia [85].

It is possible to assume that taVNS, particularly the auricular technique, can directly modulate interoception and, consequently, cardiac and respiratory interoception processes. Despite there being few experimental studies on this kind of manipulation, afferent fibers of the vagus nerve obtain information from pulmonary stretch receptors and aortic baroreceptors and convey them to the NTS [53,86]. Therefore, taVNS may be an effective way to modulate respiratory interoceptive signals, as it has been tested for cardiac interoceptive signals. Some studies have demonstrated the mechanism by which taVNS can improve the performance of heartbeat detection tasks, which extends the interoceptive accuracy level (the ability to objectively detect signals arriving from one’s own body) [87]. Villani et al. [88] reported improved accuracy during active taVNS on the heartbeat discrimination task, but not on the heartbeat counting task.

Additionally, taVNS is associated with possible modulation of gastrointestinal function [89]. Furthermore, taVNS could regulate the frequency of gastrointestinal contraction [90] and reduce gastric myoelectric frequency [91].

Moreover, some studies that used functional magnetic resonance imaging have indicated that taVNS produces significant cortical effects in the vagal afferent pathway involved in interoceptive processes, such as the NTS, thalamus, paracentral lobule, post central gyrus, and insular cortex [92,93,94]. Additionally, the paracentral lobule and insular cortex are particularly important for body representations.

### 4.4. taVNS: Parameters, Limitations and Implications

Compared to the invasive technique, taVNS is a simple, inexpensive, and safe technique commonly used in rehabilitation and clinical settings. It has minimal side effects such as headaches, pain, dizziness, and skin irritation at the stimulation site [95,96]. However, any adverse reaction, as discomfort or pain, can be monitored constantly [55,97]. An anode and a cathode are connected to the stimulation device and are placed in the inner part of the ear and in the outer part (tragus), respectively [94,97]. The presence of two electrodes paired with an intermittent stimulation (e.g., about 1 h of stimulation for three or four sessions per day) allows taVNS to intervene only on afferent fibers [73]. Moreover, it is recommended, for each stimulation session, an alternation between phases of 30 s on and 60 s off is applied, to further reduce possible adverse reactions [97]. In this regard, particular attention is needed when applying taVNS on patients with an SCI lesion above T6. They are particularly vulnerable, and could present with cardiovascular impairments [98,99]. Therefore, it would be useful to monitor patients’ heart rate while using the technique, as well as to select stimulation parameters that minimize the risk of affecting HRV. The selection of the optimal parameters is still a topic of debate and the literature data are often conflicting. However, the use of a frequency that does not exceed 25 Hz and a pulse width that is around 250 ms is recurrent [88,94]. Besides alternating on-off stimulations, SCI patients could be submitted to short sessions of taVNS not exceeding 10 min. This seems to avoid effects on HRV [100]. A recent study suggests an intermittent stimulation at parameters used for targeted neuroplasticity (30 Hz, 0.8 mA) as a potential and safe intervention after SCI [101]. Finally, taVNS can be safely used with stimulating electrodes applied to the left but not the right ear to control for cardiovascular side-effects [102]. Given the unstable blood pressure and cardiovascular consequences of SCI, and the direct implications of vagal stimulation in the modulation of autonomic tone, it is essential to better assess safety in a preliminary study.

## 5. Towards Integration with Novel Technologies: taVNS and Training with Assistive Tools

In conclusion, we want to highlight a rehabilitative perspective that aims for greater flexibility of bodily representations, considering not only the motor and sensory processes but also the potential contribution of interoception [45,103]. Interoception is essential in the experience of body awareness. In complete SCI, interoception may be the only spared sensory function that is potentially able to connect deafferented body parts with a general body representation [104]. In other words, the technique of taVNS supposedly modulates interoceptive processes [88], thus reinforcing the connection of deafferented body parts with a general body representation to promote neuroplasticity [73,74]. In a patient with brachial plexus avulsion injury, tactile stimulation by light touch to the aural territory innervated from a branch of the vagus nerve revealed the plastic reorganization of body perception [105,106]. When a phantom ear sensation vanishes, the same body changes can be experimentally re-induced by applying tactile stimulation to vagal nerves in the ear in a modified RHI paradigm [107]. Thus, taVNS may play an important role in multisensory integration that mediates the embodiment of a tool. To be able to include salient assistive tool adopting an embodied approach may enhance the potentiality of damaged body after SCI [108].

Nevertheless, taVNS could present several advantages for SCI patients. Its application during rehabilitation training with assistive tools (i.e., exoskeleton) will likely allow subjects, specifically those with more severe injuries, to feel again an “insentient” body. Whereby, the un-updating of their body representation does not occur only through visual feedback or residual afferences. To include a tool as a part of their own body, two elements are essential. First, SCI patients must use a tool that partially restores their movement [94]. However, they also need to experience a better sense of body consciousness, specifically the internal body awareness.

Using RAVANS, several studies have reported on a stronger activation of some vagal targets, such as the NTS or neuromodulation nuclei (i.e., locus coeruleus and raphe nuclei) on synchronizing the stimulation with the exhalation phase [109,110]. The coordination between the respiratory rhythm and movement could further represent an approach to rehabilitation with an assistive tool, particularly the exoskeleton [111,112]. Matching of the eyesight to the voluntarily controlled respiratory rhythm might enable the synchronization of the patient with exoskeleton movement to improve the ability of autonomous walking.

Finally, we want to emphasize the effect of this rehabilitation perspective on neuropathic or phantom pain in patients with SCI. The patients describe it as a way to restore a sense of the body, and a remaining link with it [10,113,114]. However, pain also exerts a relevant and highly disabling impact on these patients [114,115]. Moreover, it is often paired with body awareness alterations and is attributed to maladaptive plasticity [116]. The multisensory integration processes induced by virtual reality (VR) supposedly reduce the perception of pain in patients with SCI [117]. Consequently, taVNS could enhance the embodiment of a tool, alleviate pain sensations, and avoid maladaptive plasticity [25,118,119]. Furthermore, the analgesic effects of taVNS could also be applied to neuropathic pain, contributing to reduced alterations in body awareness and further improving embodiment effects.
